# Immunohistochemical Evaluation of Basal and Luminal Markers in Bladder Cancer: A Study from a Single Institution

**DOI:** 10.3390/life14121670

**Published:** 2024-12-17

**Authors:** Anh Toan Do, Quoc Thang Pham, Ngoc Minh Tam Nguyen, Phuc Nguyen Nguyen, Thi Thanh Tam Bui, Quoc Dat Ngo

**Affiliations:** 1 Department of Urology, University of Medicine and Pharmacy at Ho Chi Minh City, Ho Chi Minh City 70000, Vietnam; doanhtoan@ump.edu.vn; 2 Department of Pathology, University of Medicine and Pharmacy at Ho Chi Minh City, Ho Chi Minh City 70000, Vietnam; buithithanhtamyds@ump.edu.vn (T.T.T.B.); ngoquocdat@ump.edu.vn (Q.D.N.); 3 Department of Pathology, Binh Dan Hospital, Ho Chi Minh City 70000, Vietnam; bstamgpb@gmail.com; 4 Department of Oncology, Binh Dan Hospital, Ho Chi Minh City 70000, Vietnam; nguyenphucnguyen.vn@gmail.com

**Keywords:** bladder cancer, immunohistochemistry, molecular subtypes, tumor-infiltrating lymphocytes

## Abstract

Background: Bladder cancer (BC) presents significant molecular diversity, which affects both prognosis and treatment results. Immunohistochemistry (IHC) facilitates the identification of molecular subtypes and their relationships with clinicopathological features. Methods: We performed an IHC analysis on tissue samples from 107 BC patients, evaluating the expression of markers GATA3, CD44, CK5/6, and CK20. We applied two methods to classify the tumor samples into basal and luminal subtypes. The relationships between these marker expressions, molecular subtypes, clinicopathological characteristics, and TILs were explored. Results: Most samples showed the expression of GATA3 and CD44, with notable correlations found between CD44 and CK5/6 as well as GATA3 and CK20. CD44 and CD20 expression were linked to a poorer prognosis. Additionally, the luminal and basal subtypes had distinct TIL patterns, which influenced overall survival. A poor prognosis was associated with the basal subtype with low TIL infiltration and the luminal subtype with high TIL infiltration. Conclusions: Our study clarifies the molecular characteristics of BC, underlining the prognostic importance of CD44 expression and the role of TILs in influencing subtype-specific outcomes. IHC proves valuable in subtype identification and supports personalized treatment strategies.

## 1. Introduction

Bladder cancer (BC) ranks as the 11th most common cancer globally, contributing significantly to mortality rates worldwide [1]. In Vietnam, bladder cancer cases are expected to increase by 80.6% by 2045 [2]. Urothelial bladder cancer is one of the most challenging and expensive cancers to diagnose and manage. Among those diagnosed, the majority present with non-muscle invasive bladder cancer (NMIBC), representing early-stage disease, while approximately 25% are diagnosed with muscle-invasive bladder cancer (MIBC) [3]. Standard treatment options for bladder cancer include transurethral resection followed by intravesical chemotherapy or BCG for NMIBC and cystectomy for more advanced stages [4]. The prognosis for patients with MIBC remains poor [4]. Thus, there is an unmet need for predicting urothelial bladder cancer overall survival and for personalizing treatments.

Many studies have sought to uncover the link between genomic tumor profiles, histopathological patterns, and clinical outcomes. The Cancer Genome Atlas (TCGA) project identified two main subtypes of MIBC: basal and luminal [5]. NMIBC has been classified into basal- and luminal-like subtypes, each with different prognoses [6]. Additionally, basal tumors showed significant improvement in overall survival when treated with neoadjuvant therapy, while luminal tumors have generally been associated with more favorable outcomes than basal tumors [7,8].

Most of these molecular subtypes are identified through gene expression profiling. However, recent advances have demonstrated that immunohistochemical (IHC) markers, either used individually or in panels, can assist pathologists and oncologists in categorizing bladder tumors into luminal or basal subtypes [9,10]. In this study, we utilized a panel of IHC markers to classify bladder cancer subtypes and examined the correlations between these subtypes and clinicopathological features.

## 2. Materials and Methods

### 2.1. Tissue Samples

We conducted a retrospective study involving primary tumors from 107 patients diagnosed with urothelial carcinoma who underwent radical cystectomy at Binh Dan Hospital in Ho Chi Minh City, Vietnam (from 2017 to 2018). After the cystectomy, patient follow-up was tracked through medical records. Clinical data were collected from the patients’ pathology reports. A detailed review of Hematoxylin and Eosin (H&E) sections was performed to evaluate pathologic characteristics, including lymphovascular invasion, perineural invasion, tumoral necrosis, squamous differentiation, glandular differentiation, the presence of tumor-infiltrating lymphocytes (TILs), and the pathological stage according to the AJCC TNM classification system (8th edition) [11].

TILs were evaluated on H&E sections based on recommendations made by the International TILs Working Group in 2014 [12]. More than 10% of lymphocyte infiltration was considered to be an intense TIL, whereas less than 10% infiltration was considered to be a non-intense TIL [13].

Patients who had received neoadjuvant chemotherapy or preoperative BCG treatment were excluded from the study. Informed consent was obtained from each participant, and the study was approved by the Ethics Committee at the University of Medicine and Pharmacy in Ho Chi Minh City (approval number 820/HĐĐĐ-ĐHYD).

### 2.2. Immunohistochemistry and Evaluation

The immunohistochemical (IHC) staining was performed on 5 µm thick tissue sections. Initially, the sections were incubated for one hour at room temperature with the primary antibodies. Following this, the sections were treated for another hour using the Dako Envision+ Peroxidase Detection System (Dako Cytomation, Carpinteria, CA, USA), with anti-mouse or anti-rabbit secondary antibodies. The color reaction was developed using the Dako Cytomation DAB Substrate-Chromogen Solution. The primary antibodies used in this study included: mouse Cytokeratin 5/6 monoclonal antibody (1:50; catalog no. MA1-91106, Invitrogen/ThermoFisher Scientific, Waltham, MA, USA); mouse Cytokeratin 20 monoclonal antibody (1:50; catalog no. MA5-13263, Invitrogen/ThermoFisher Scientific, Waltham, MA, USA); mouse GATA3 monoclonal antibody (1:200; catalog no. MA1-028, Invitrogen/ThermoFisher Scientific, Waltham, MA, USA); and mouse CD44 monoclonal antibody (1:200; catalog no. MA5-13890, Invitrogen/ThermoFisher Scientific, Waltham, MA, USA).

The expression of these markers was assessed using an expression score, which considered both intensity (rated as 1+, 2+, and 3+) and the percentage (ranging from 0 to 100%) of positive tumor cells (Figure S1) [14]. Two surgical pathologists (Q.D.N. and M.T.N.) independently evaluated the IHC results, blinded to any clinicopathologic data and patient outcomes.

We employed two methods to classify the tumor samples into basal and luminal subtypes. In the 1st method, the subtype classification was based on the marker with the highest expression score, using a two-step cluster analysis for comparison (Figure S2). In the 2nd method, samples were categorized into four types: basal type: positive for CK5/6 and/or CD44 and negative for both CK20 and GATA3; luminal type: negative for CK5/6 and CD44 and positive for either CK20 or GATA3; null type: negative for all four markers; and mixed type: expressing both basal and luminal markers. Tumor cells were considered positive if the target protein expression was greater than 20% of cells. For GATA3, positivity was based on nuclear staining, while for CD44, CK5, and CK20, positivity was based on membrane/cytoplasmic staining [15].

### 2.3. Analysis of TCGA Data

The BLCA-TCGA cohort, publicly available data were analyzed for markers expression and survival analysis (Broad GDAC Firehose, https://xenabrowser.net/, accessed on 20 June 2024) [16,17].

### 2.4. Statistical Methods

The relationship between clinicopathological features and IHC markers expression were evaluated by χ2 test. Spearman rank correlation was applied to analyze the correlation between marker IHC expression. Kaplan–Meier survival curves were constructed for patients with basal or luminal subtype of BC. Survival rates were compared between the basal or luminal subtype of BC. Differences between survival curves were tested for statistical significance by a log-rank test. All statistical analyses were performed using SPSS ver. 20.0 (Chicago, IL, USA).

## 3. Results

### 3.1. Characteristics of Patients

Our study included 107 bladder cancer (BC) patients, with the vast majority being male (89.7%). The average age at diagnosis was 62 years, with patients ranging in age from 30 to 89 years. Additional information on the clinicopathologic characteristics of the cohort is provided in Supplementary Table S1.

### 3.2. Correlation Between Marker Expression and Clinicopathologic Features

**Figure 1:**
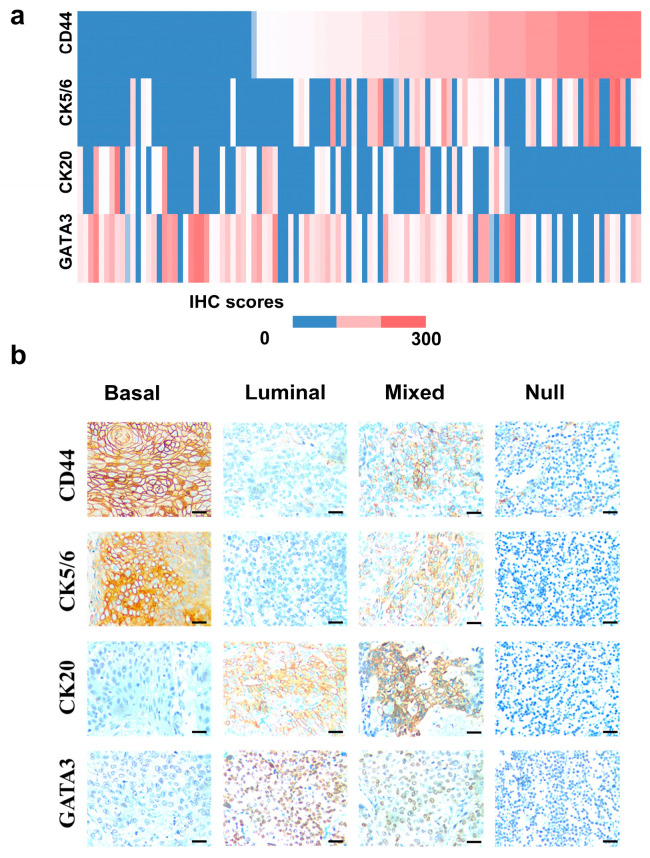
The immunohistochemistry expression of CD44, CK5/6, CK20, and GATA3. (**a**) The heatmap presented the immunoreactive of fours markers; (**b**) IHC stained with CD44, CK5/6, CK20, and GATA3 presenting representative molecular subtypes as basal, luminal, mixed, and null-types, the scale bar 50 μm.

As shown in Figure 1, most cases exhibited immunoreactivity with GATA3 (81.4%) and CD44 (69.2%). In contrast, CK5/6 (43%) and CK20 (46.4%) showed more variable expressions across bladder cancer samples. A significant correlation was observed between CD44 and CK5/6 expression (rho = 0.55, *p* < 0.001) and between GATA3 and CK20 expression (rho = 0.38, *p* < 0.001) (Figure S3).

The association between marker expression and clinicopathologic features of bladder cancer is detailed in Table 1. No significant associations were found between the expression of CD44, CK5/6, GATA3, or CK20 and parameters such as stage, vascular invasion, or neural invasion. However, GATA3 (luminal marker) and CK5/6 (basal marker) were significantly correlated with squamous differentiation and high levels of tumor-infiltrating lymphocytes (TILs). Additionally, the basal marker CD44 was strongly associated with squamous differentiation. Moreover, both our data and the TCGA BLCA database revealed that patients positive for CD44 had poorer overall survival and disease-free survival (Figure S4a,b).

### 3.3. Evaluating Basal and Luminal Subtypes Correlated with Overall Survival

We explored the relationship between basal and luminal subtypes and various clinicopathologic features, along with the clinical outcomes of bladder cancer (BC) patients. Our findings indicated a strong association between molecular subtypes and squamous differentiation and tumor-infiltrating lymphocytes (TILs), regardless of the classification method employed (Supplementary Table S2 and Table 2). However, our analysis showed no significant correlation between molecular subtypes and the tumor stage or other pathological characteristics, such as tumoral necrosis, lymphovascular invasion, or neural invasion (Supplementary Table S2 and Table 2).

As presented in Table 3, the univariate and multivariate Cox regression analysis identified CD44 and CK20 as significant prognostic markers for predicting BC outcomes. However, the molecular subtypes themselves did not show a significant association with BC prognosis.

### 3.4. TILs and Basal and Luminal Subtypes Affect Patient Outcomes

**Figure 2:**
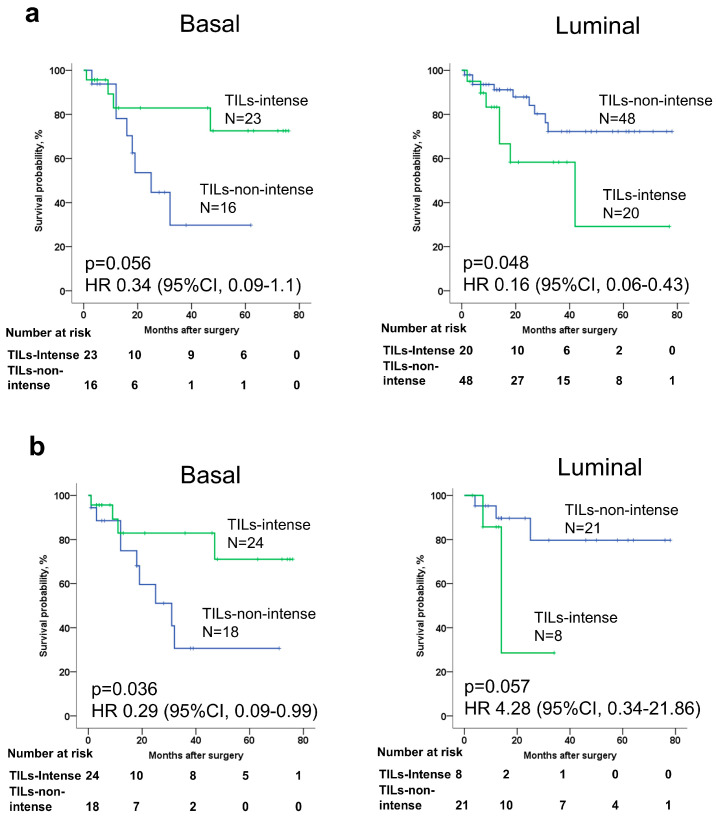
Basal and luminal tumors intergated with TILs, associated with BC patient outcomes. (**a**) Survival analysis of BC patients with intense and non-intense TILs in association with basal and luminal subtype BC defined in 1st method; (**b**) survival analysis of BC patients with intense and non-intense TILs in association with basal and luminal molecular subtype defined in 2nd method.

We found that the majority of luminal subtypes showed a non-intensive pattern of tumor-infiltrating lymphocytes (TILs) (Supplementary Table S2 and Table 2). In contrast, the basal subtype was more commonly associated with an intensive TIL pattern. To explore this further, we analyzed the relationship between TIL patterns and molecular subtypes in relation to overall survival. Our results indicated that basal subtypes with non-intensive TILs had a worse prognosis compared to basal subtypes with intensive TILs (Figure 2a,b). Interestingly, for the luminal subtype, patients with non-intensive TILs demonstrated a better prognosis compared to those with intensive TILs (Figure 2a,b).

## 4. Discussion

In recent years, the identification of molecular subtypes in bladder cancer has gained significant attention due to its impact on prognosis and treatment response. There is now substantial evidence supporting the view that bladder cancer is not a singular disease but rather a collection of molecularly distinct subtypes, each with unique biological behaviors and clinical outcomes. Various RNA sequencing methods have been employed to classify these molecular subtypes, including the MDA, Lund, and TCGA classification [5,9,18]. While these classifications differ, the foundational subtypes identified are consistently the basal and luminal subtypes. Luminal subtypes are characterized by the high expression of genes like GATA3 and KRT20, whereas basal subtypes show the upregulation of markers such as CD44, KRT14, KRT5, and KRT6B [9,18]. Several studies have suggested using a combination of luminal and basal protein markers, including GATA3, FOXA1, KRT5/6, and KRT14 [9,19]. Our findings, in conjunction with TCGA data, highlight that CD44 expression is linked to poorer outcomes in bladder cancer. Additionally, our study shows that the significant co-expression of basal markers (CD44, CK5/6) or luminal markers (GATA3, CK20) as well as cases with negative expression for both marker sets can effectively classify the subtypes. Thus, using these four markers in IHC provides a reliable tool for distinguishing between basal and luminal subtypes of bladder cancer.

Tumor-infiltrating lymphocytes (TILs) are crucial in influencing both tumor progression and treatment response across various cancers, including bladder cancer. Higher TIL levels have been associated with improved survival outcomes, particularly in patients with muscle-invasive bladder cancer (MIBC) who received neoadjuvant therapy [20,21]. In a multicenter retrospective study, basal tumor bladder cancer patients had worse survival outcomes when treated without neoadjuvant therapy compared to those with luminal tumors [22]. However, patients with basal-like tumors showed the greatest benefit from neoadjuvant therapy [8].

In our study, we observed that intense TILs were strongly correlated with a better prognosis, especially in basal subtype tumors. Conversely, luminal-infiltrated bladder cancer has been reported to have a poor prognosis regardless of whether neoadjuvant chemotherapy was administered. This highlights the varying impact of TILs on different molecular subtypes of bladder cancer and their potential role as a prognostic factor [8,9,23].

Our study has limitations. With the retrospective collected sample, our data included a small number of NMIBC (*n* = 7). This study aimed to classify bladder cancer subtypes using a panel of IHC markers (CK5/6 and CD44 for basal; GATA3 and CK20 for luminal) widely used in surgical pathology. However, NMIBC and MIBC were not analyzed separately, which may have obscured subtype-specific differences. Future studies will address this limitation by focusing on distinct NMIBC and MIBC cohorts.

## 5. Conclusions

Our research employed IHC to detect the expression of key markers associated with basal and luminal subtypes, specifically CD44, CK5/6 (basal markers), and GATA3, CK20 (luminal markers). Our study sheds light on the intricate interplay between molecular subtypes and tumor-infiltrating lymphocytes (TILs) in bladder cancer. We observed distinct expression patterns of basal and luminal markers, emphasizing their relevance in determining prognosis and treatment response. Notably, CD44 expression emerged as a significant prognostic indicator, correlating with poorer outcomes in bladder cancer patients. Furthermore, our findings underscore the impact of TILs on patient survival, particularly in basal subtype tumors, where intense TILs correlate with an improved prognosis.

## Figures and Tables

**Table 1:** The association between positive marker expression and clinicopathologic features of BC patients.

	Makers			
	CD44 N (%)	CK5/6 N (%)	CK20 N (%)	GATA3 N (%)
Histological grade				
Low grade	16 (53.3)	4 (13.3)	7 (23.03)	15 (50.0)
High grade	44 (57.1)	25 (32.5) *	16 (20.8)	29 (37.7)
Stage				
I	6 (85.7)	2 (28.6)	3 (42.9)	4 (57.1)
II	26 (55.3)	14 (29.8)	10 (21.3)	20 (42.6)
III	16 (48.5)	7 (21.2)	6 (18.2)	14 (42.4)
IV	12 (60.0)	6 (30.0)	4 (20.0)	6 (30.0)
Squamous differentiation				
No	44 (51.2)	15 (17.4)	21 (24.4)	40 (46.5)
Yes	16 (76.2) *	14 (66.7) *	2 (9.5)	4 (19.0) *
Tumoral necrosis				
No	18 (54.5)	11 (33.3)	10 (30.3)	14 (42.4)
Yes	42 (56.8)	18 (24.3)	13 (17.6)	30 (40.5)
TILs				
Non-intense	31 (48.4)	7 (10.9)	14 (21.9)	32 (50.0)
Intense	29 (67.4)	21 (51.2) *	9 (20.9)	23 (37.1) *
Vascular invasion				
No	37 (59.7)	17 (27.4)	11 (17.7)	21 (46.7)
Yes	23 (51.1)	12 (26.7)	12 (26.7)	4 (80)
Neural invasion				
No	45 (55.6)	23 (28.4)	20 (24.7)	33 (40.7)
Yes	15 (57.7)	6 (23.1)	3 (11.5)	11 (42.3)

* *p* < 0.05 when comparing the positive with negative group.

**Table 2:** The relationship between molecular subtypes classified by the 2nd method and the clinicopathologic features of BC patients.

		Molecular Subtypes				*p*-Value
		Basal N (%)	Lunimal N (%)	Null N (%)	Mix N (%)	
Histological grade	Low grade	10 (33.3)	9 (30.0)	4 (13.3)	7 (23.3)	0.793
	High grade	32 (41.6)	20 (26.0)	12 (15.6)	13 (16.9)	
Stage	Stage I	2 (28.6)	0	1 (14.3)	4 (57.1)	
	II	19 (40.4)	15 (31.9)	5 (10.6)	8 (17.0)	
	III	10 (30.3)	10 (30.3)	7 (21.20	6 (18.2)	0.240
	IV	11 (55.0)	4 (20.0)	3 (15.0)	2 (10.0)	
Squamous differentiation	No	26 (30.2)	25 (29.1)	15 (17.4)	20 (23.3)	0.001
	Yes	16 (76.2)	4 (19.0)	1 (4.8)	0	
Tumoral necrosis	No	12 (36.4)	8 (24.2)	6 (18.2)	7 (21.2)	0.856
	Yes	30 (40.5)	21 (28.4)	10 (13.5)	13 (17.6)	
Peritumoral inflammation	No	18 (28.1)	21 (32.8)	11 (17.2)	14 (21.9)	0.042
	Yes	24 (55.8)	8 (18.6)	5 (11.6)	6 (14.0)	
Vascular invasion	No	26 (41.9)	13 (21.0)	10 (16.1)	13 (21.0)	0.436
	Yes	16 (35.6)	16 (35.6)	6 (13.3)	7 (15.6)	
Neural invasion	No	32 (39.5)	22 (27.2)	12 (14.8)	15 (18.5)	1
	Yes	10 (38.5)	7 (26.9)	4 (15.4)	5 (19.2)	

**Table 3:** Univariate and multivariate Cox regression analysis of clinicopathologic features and survival in bladder cancer.

		Univariate Analysis		Multivariate Analysis	
Characteristic		HR (95% CI)	*p*-Value	HR (95% CI)	*p*-Value
CD44	Negative	1 (Ref.)		1 (Ref.)	
	Positive	2.54 (1.07–5.98)	0.033	2.64 (1.12–6.25)	0.027
CK5/6	Negative	1 (Ref.)			
	Positive	0.89 (0.39–2.04)	0.789		
CD20	Negative	1 (Ref.)		1 (Ref.)	
	Positive	2.32 (1.06–5.06)	0.035	2.43 (1.12–5.32)	0.026
GATA3	Negative	1 (Ref.)			
	Positive	0.74 (0.34–1.61)	0.453		
Molecular subtypes **	Basal	1			
	Luminal	0.64 (0.24–1.69)	0.371		
	Negative	0.16 (0.02–1.20)	0.074		
	Mix	1.09 (0.45–2.53)	0.853		
Molecular subtypes *	Basal	1 (Ref.)			
	Luminal	0.69 (0.33–1.47)	0.339		

* Classified by 1st method; ** classified by 2nd method HR: hazard ratio, CI: confidence interval.

## Data Availability

All data generated or analyzed during this study are included in this published article. Any other queries about the data used in this study should be directed to the corresponding author.

## Supplementary Materials

The following supporting information can be downloaded at: https://www.mdpi.com/article/10.3390/life14121670/s1, Figure S1. The expression of markers expressed with the intensity (1+, 2+, 3+). Figure S2. Two-step Cluster method classified sample as a basal or luminal category. Figure S3. Correlation of CD44, CK5/6, CK20 and GATA3 Expression in Bladder Cancer. Figure S4. (a) Overal survival analysis of BC patients with positive and negative markers expression by Kaplan-Meier method. (b) Disease specific survival analysis of BC patients in BLCA data set with high and low markers expression by Kaplan-Meier method. Table S1. Clinicopathologic features of bladder cancer patients. Table S2. The association between basal and luminal subtypes classified by the 1st method and the clinicopathologic features of BC patients.
