# Supplementary material for: Immunohistochemical Evaluation of Basal and Luminal Markers in Bladder Cancer: A Study from a Single Institution
Source: Life (Basel). 2024 Dec 17;14(12):1670. doi: 10.3390/life14121670 (PMC11728301; doi:10.3390/life14121670)

Supplementary Table S1. Clinicopathologic features of bladder cancer patients.

Features		N (%)
Age	< 60	38 (35.5)
	≥ 60	69 (64.5)
Sex	Female	11 (10.3)
	Male	96 (89.7)
Histological grade	Low grade	30 (28.0)
	High grade	77 (72.0)
Stage	I	7 (6.5)
	II	47 (43.9)
	III	33 (30.8)
	IV	20 (18.7)
Squamous differentiation	No	86 (80.4)
	Yes	21 (19.6)
Glandular differentiation	No	104 (97.2)
	Yes	3 (2.8)
Tumoral necrosis	No	13 (12.1)
	Yes	94 (87.9)
TILs	Non-intense	64 (59.8)
	Intense	43 (40.2)
Vascular invasion	No	62 (57.9)
	Yes	45 (42.1)
Neural invasion	No	81 (75.7)
	Yes	26 (24.3)

Tumor-Infiltrating Lymphocytes (TILs)

Supplementary Table S2. The association between basal and luminal subtypes classified by the 1st method and the clinicopathologic features of BC patients

		Molecular subtypes		p value
		Basal<br>N (%)	Lunimal<br>N (%)	
Histological grade	Low grade	7 (23.3%)	23	0.078
	High grade	32 (41.6%)	45	
Stage	I	1 (14.3%)	6	0.126
	II	18 (38.3%)	29	
	III	9 (27.3%)	24	
	IV	11 (55%)	9	
Squamous differentiation	No	23 (26.7%)	63	<0.0001
	Yes	16 (76.2%)	5	
Glandular differentiation	No	39 (37.5%)	65	0.299
	Yes	0	3	
Tumoral necrosis	No	13 (39.4%)	20	0.672
	Yes	26 (35.1%)	48	
TILs	Non-intense	16 (25%)	48	0.003
	Intense	23 (53.5%)	20	
Vascular invasion	No	23 (37.1%)	39	0.87
	Yes	16 (35.6%)	29	
Neural invasion	No	28 (34.6%)	53	0.476
	Yes	11 (42.3%)	15	

Figure S1. The expression of markers expressed with the intensity (1+, 2+, 3+)

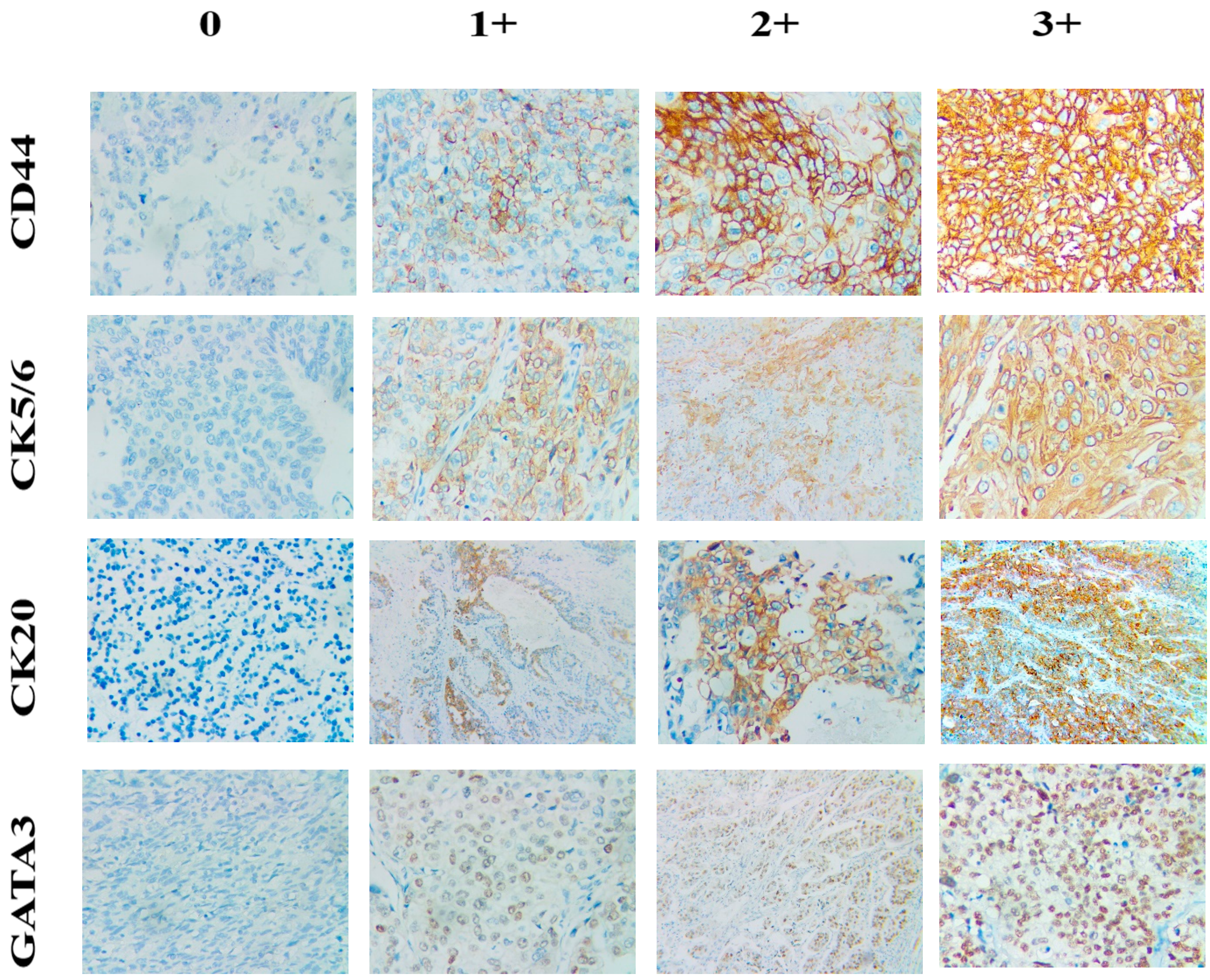

Figure S2. Two-step Cluster method classified sample as a basal or luminal category.

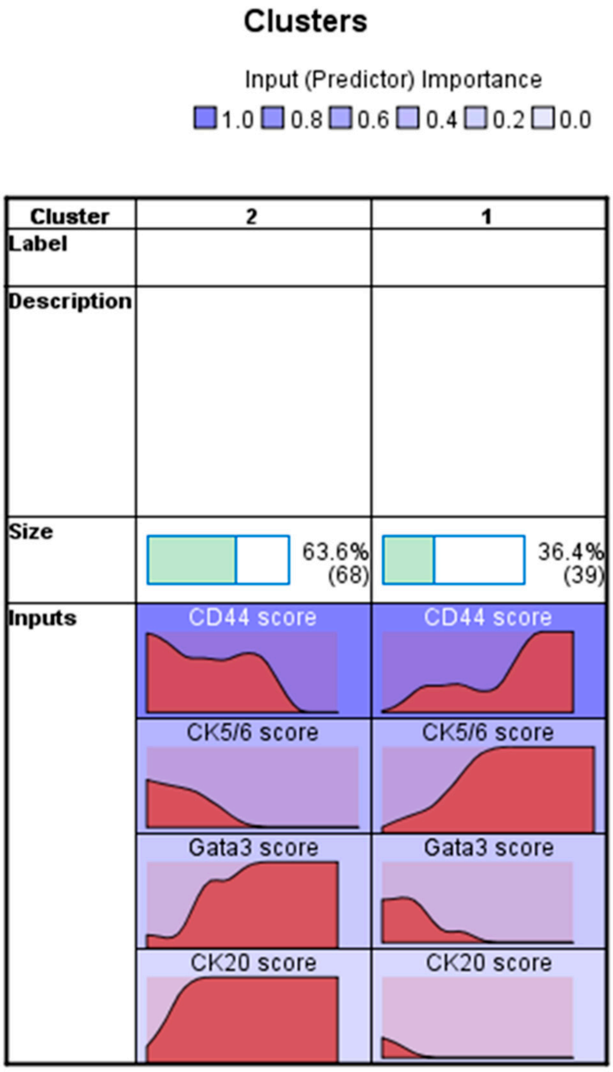

Figure S3. Correlation of CD44, CK5/6, CK20 and GATA3 Expression in Bladder Cancer

	CD44	CK5/6	CK20	GATA3
CD44	1	0,55**	-0,29*	-0,27*
CK5/6	0,55**	1	-0,28*	-0,28*
CK20	-0,29*	-0,28*	1	0,38**
GATA3	-0,27*	-0,28*	0,38**	1

Spearman's rho

**p<0.001; *p<0.01

Figure S4. (a) Overal survival analysis of BC patients with positive and negative markers expression by Kaplan-Meier method. (b) Disease specific survival analysis of BC patients in BLCA data set with high and low markers expression by Kaplan-Meier method.

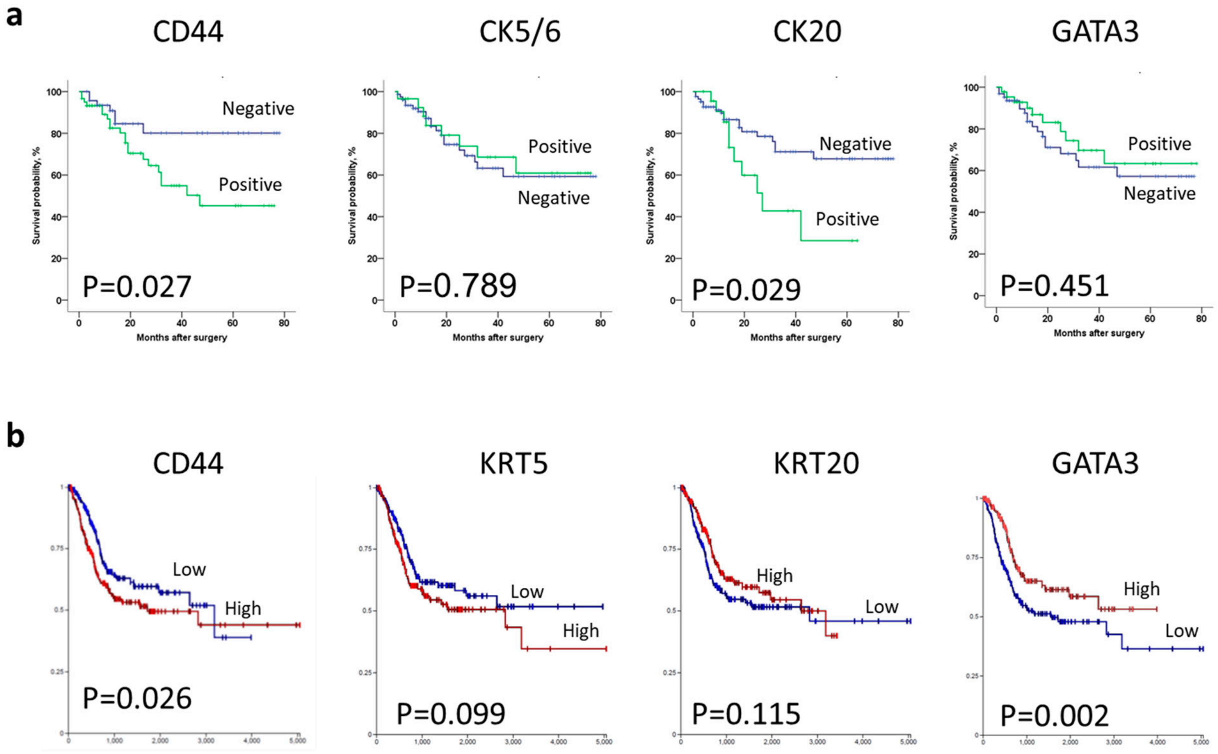

Supplement: Supplementary file 1 [file life-14-01670-s001.zip › life-3313023-supplementary.pdf]
